# Health-related quality of life correlates with patient-reported and proxy-reported disability in critical illness survivors: a secondary analysis of the ERIC trial

**DOI:** 10.1186/s13054-025-05399-3

**Published:** 2025-04-23

**Authors:** Elena Ribet Buse, Julius J. Grunow, Claudia D. Spies, Björn Weiss, Nicolas Paul

**Affiliations:** Department of Anesthesiology and Intensive Care Medicine (CCM/CVK), Charité – Universitätsmedizin Berlin, corporate member of Freie Universität Berlin and Humboldt-Universität zu Berlin, Augustenburger Platz 1, 13353 Berlin, Germany

**Keywords:** Critical care, Disability, EQ-5D-5L, Health-related quality of life, Post-ICU care, Post-intensive care syndrome, WHODAS

## Abstract

**Background:**

Expanding follow-up services for survivors of critical illness requires short and reliable instrument sets. The WHO Disability Assessment Schedule (WHODAS) 2.0 and the EuroQol 5-Dimensions 5-Level (EQ-5D-5L) are recommended to assess disability and health-related quality of life (HrQoL), respectively. As they may measure partially overlapping constructs, we assessed their relationship.

**Methods:**

We conducted a secondary analysis of the multicenter cluster-randomized controlled Enhanced Recovery after Intensive Care (ERIC) trial (ClinicalTrials.gov: NCT03671447). At follow-ups scheduled 6 months after ICU discharge, critical illness survivors and caregivers completed the EQ-5D-5L, the patient-reported and the proxy-reported 12-item WHODAS 2.0. We employed local polynomial regressions, correlation coefficients, and linear regressions to analyze the global and domain-specific relationships between the EQ-5D-5L and the WHODAS 2.0.

**Results:**

We analyzed 700 patients with a median EQ-5D-5L index value of 0.81 [IQR 0.52 to 0.94], a median patient-reported WHODAS 2.0 sum score of 11 [IQR 3 to 23], and a median proxy-reported WHODAS 2.0 sum score of 16 [IQR 6 to 28]. The EQ-5D-5L index value highly correlated with patient-reported (Spearman: − 0.84 [95% CI − 0.86 to − 0.82]) and proxy-reported (Spearman: − 0.70 [− 0.76 to − 0.64]) WHODAS 2.0 sum scores. Corresponding domains were also highly correlated, with the patient-reported WHODAS 2.0 aligning more closely with the EQ-5D-5L than the proxy-reported WHODAS 2.0. We found ceiling and floor effects for both instruments, indicating limitations for detecting mild disabilities and high HrQoL. In multivariable linear regressions, the patient-reported and proxy-reported WHODAS 2.0 sum scores (both − 0.02 [95% CI − 0.02 to − 0.02], *p* < 0.01) and WHODAS 2.0 domain scores for mobility, self-care, and life activities were predictors of the EQ-5D-5L index value and respective EQ-5D-5L domain scores.

**Conclusions:**

Our results suggest a high correlation between the patient-reported and proxy-reported WHODAS 2.0 and the EQ-5D-5L, particularly in their corresponding domains. To economize post-ICU assessments, there may be no need to use both instruments simultaneously.

**Supplementary Information:**

The online version contains supplementary material available at 10.1186/s13054-025-05399-3.

## Introduction

As survival rates of critically ill patients admitted to an intensive care unit (ICU) increase [1], understanding the challenges ICU survivors face during the recovery trajectory becomes increasingly vital [2]. These patients are burdened by new or worsened physical, mental, and cognitive impairments, which is summarized as post-intensive care syndrome (PICS) [3]. Follow-up studies have revealed that PICS leads to increased disability [4] and diminishes ICU survivors’ health-related quality of life (HrQoL) [5–7].

Survivors of critical illness often receive post-ICU care by their primary care physician [8] or in post-ICU follow-up clinics [9–11]. Irrespective of their follow-up location, international consensus statements unanimously recommend assessments with validated instruments [12, 13], which should be quick and easy to complete, considering the high morbidity of these patients [12]. However, post-ICU programs and long-term outcome studies struggle with high no-show rates and loss to follow-up [9, 14]. Especially severely impaired individuals unable to attend in-person appointments could be underrepresented in ICU follow-up programs, although they could benefit from those [15]. Avoiding duplicate instruments, reducing the time required for follow-up assessments, and using instruments that can be flexibly administered in person, via telephone, or via mail may reduce the burden for patients and reduce selection bias of follow-up programs.

The EuroQol 5-Dimensions 5-Level (EQ-5D-5L) [16–18] and the World Health Organization Disability Assessment Schedule 2.0 (WHODAS 2.0) [19] are recommended for assessing HrQoL and disability, respectively [12]. The EQ-5D-5L correlates well with subjective health [20, 21] and both instruments have been validated among ICU survivors [22, 23]. For the WHODAS 2.0, patient-reported and proxy-reported versions exist [24, 25]. The domains of both instruments partially overlap, namely mobility, self-care and life activities. Hence, it is unclear whether the simultaneous use of the WHODAS 2.0 and the EQ-5D-5L in ICU survivors is meaningful. Among one cohort of ICU survivors in Australia, the global Spearman correlation coefficients of the patient-reported WHODAS 2.0 and the EQ-5D-5L visual analogue scale (VAS) and EQ-5D-5L index value were − 0.72 and − 0.81, respectively [22, 23]. However, determining the added value of the simultaneous use of the WHODAS 2.0 and EQ-5D-5L may also require an analysis of overlapping domains of both instruments and whether one instrument can be used to derive the other. Also, only the patient-reported version of the WHODAS 2.0 has been previously analyzed [22, 23]. Although the proxy-reported WHODAS 2.0 may be particularly valuable among ICU survivors unable to fill out the patient-reported version, neither the correlation of the proxy-reported WHODAS 2.0 with the EQ-5D-5L nor the agreement of the patient-reported and proxy-reported WHODAS 2.0 have been analyzed for ICU survivors.

Firstly, this study explores the relationship between the EQ-5D-5L and the 12-item WHODAS 2.0 among a German cohort of ICU survivors, analyzing the relationship of overlapping domains of both instruments. We separately analyze the relationship of the patient-reported and the proxy-reported WHODAS 2.0 with the EQ-5D-5L. Finally, the relationship between the patient-reported and proxy-reported WHODAS 2.0 is analyzed.

## Methods

### Study design, setting, and study population

We conducted a secondary analysis of data from the multicenter, stepped-wedge cluster-randomized controlled Enhanced Recovery after Intensive Care (ERIC) trial (ClinicalTrials.gov: NCT03671447) [26, 27]. ERIC received ethical approval from Charité’s Institutional Review Board (EA1/006/18) on January 26, 2018. Patients were enrolled in ten clusters of ICUs within the metropolitan area of Berlin, Germany, between September 4, 2018, and March 31, 2020. Inclusion criteria were as follows: anticipated ICU length of stay ≥ 24 h within a mixed, medical, or surgical ICU, age ≥ 18 years, and statutory health insurance coverage. Informed consent was obtained from patients or their legal representatives. For the purpose of this secondary analysis, we included patients who were discharged alive from the ICU and completed the EQ-5D-5L as well as the patient-reported and/or proxy-reported 12-item WHODAS 2.0 at the follow-up assessment scheduled after 6 months.

### Post-ICU follow-ups

All patients were scheduled for two follow-up assessments at three and 6 months after ICU discharge. The follow-ups were conducted either at the study site or as home visits by trained study personnel and followed a previously published PICS instruments set, [12] which is described in Table S1. If in-person assessments were impractical due to logistical or health-related considerations, few follow-ups were conducted via telephone or mail. For this analysis, we included the EQ-5D-5L and WHODAS 2.0 data from the 6-month follow-ups.

### The 12-item WHODAS 2.0 and EQ-5D-5L

The patient-reported and proxy-reported 12-item WHODAS 2.0 were used to assess disability [19]. In case a proxy was present at the follow-up assessment, the proxy-reported WHODAS 2.0 was completed in addition to the patient-reported version. If a patient was alive but unable to complete the patient-reported WHODAS 2.0 (e.g. in case of severe stroke) and a proxy was present at the follow-up assessment, only the proxy-reported WHODAS 2.0 was completed. The WHODAS 2.0 covers the domains of cognition, mobility, self-care, getting along, life activities, and participation. Each domain is assessed with two items. Patients rate each item as either ‘none’ (0), ‘mild’ (1), ‘moderate’ (2), ‘severe’ (3), or ‘extreme or cannot do’ (4) in the previous 30 days [19]. All items are added up to a sum score, which ranges from 0 (no disability) to 48 (severe disability) and is also expressed as a percentage of the maximum score (sum score*100/48) [19]. We also computed sum scores for each domain, ranging from 0 (no disability) to 8 (severe disability). As suggested in the manual [19], a single missing value was replaced by the mean of the remaining items. If more than one item was missing, no sum score was calculated and the patient was excluded from the analysis.

The HrQoL was assessed using the EQ-5D-5L, which consists of five items covering the dimensions mobility, self-care, usual/life activities, pain/discomfort, and anxiety/depression [16, 18]. Participants rate each item from ‘no problems’ (1) to ‘extreme problems’ (5) [17]. Responses are converted to country-specific index values (1: full health; 0: death; below 0: worse than death). We used the German value set to calculate EQ-5D-5L index values, ranging from 1 to − 0.661 [28]. Additionally, participants rate their health on a VAS from 0 to 100 points [16, 18]. Notably, higher index values and higher VAS scores signify better HrQoL, but higher scores in the EQ-5D-5L domains indicate greater impairments. WHODAS 2.0 and EQ-5D-5L share the following domains: mobility, self-care, and life activities.

### Statistical analysis

Categorical variables are presented as absolute frequencies and percentages. Continuous variables are summarized using the median and the limits of the interquartile range. Relationships between the instruments and their corresponding domains were visualized using histograms and scatterplots with locally estimated scatterplot smoothing (LOESS) curves. We computed radar charts to visualize patterns of EQ-5D-5L responses by WHODAS 2.0 sum scores, which were divided into five equal groups (sum scores 0–7, 8–15, 16–23, 24–31, 32–39, and 40–48). The relationships between the instruments and domains were analyzed using Spearman's correlation coefficient. A correlation of 0.1 to 0.39 was considered weak, 0.40 to 0.69 moderate, 0.7 to 0.89 strong, and ≥ 0.90 very strong [29]. Following previous studies, we analyzed if > 15% of participants scored within the top or bottom 20% of the respective scale, which was defined as a significant ceiling or floor effect [30]. Additionally, we report the proportion of participants with the maximum or minimum scores. To analyze if the WHODAS 2.0 responses may be used to derive the EQ-5D-5L, we computed simple linear regression models using the EQ-5D-5L index value or the EQ-5D-5L VAS as dependent variables and the patient-reported or proxy-reported WHODAS 2.0 sum score as independent variable. In additional simple linear regressions, the EQ-5D-5L domains mobility, self-care, and life activity were used as dependent variables and the respective patient-reported or proxy-reported WHODAS 2.0 domain sum scores as independent variables. In additional multivariable regressions, the following covariables were added to the previously mentioned models: age, sex, ICU length of stay (days), mechanical ventilation (hours), SAPS II on admission, and delirium during ICU stay (yes/no; measured daily with either the Confusion Assessment Method for the ICU or the Nursing Delirium Screening Checklist). As potential confounders, these covariables may have impacted the WHODAS 2.0 and EQ-5D-5L responses. Statistical analyses were performed using R (version 4.2.2, R Core Team, 2022) and RStudio (Posit Team, 2023), using the tidyverse (Wickham, 2019), ggplot2 (Wickham, 2016), eq5d (Morton, 2022), fmsb (Nakazawa, 2023), corrplot (Wei & Simko, 2021), and stargazer packages (Hlavac, 2022).

## Results

### Study population

Of 1463 patients enrolled in the ERIC trial, 158 patients died in the ICU, 1 patient was excluded during the ICU stay, and 254 before the 6-month follow-up, leaving 1050 patients. Of those, 700 patients were included in this analysis. The patient-reported WHODAS 2.0 was available for 690 patients and the proxy-reported WHODAS 2.0 for 290 patients. For 10 patients, only the proxy-reported WHODAS 2.0 was available (Fig. S1, Tables 1, S2).

**Table 1:** Baseline characteristics of the study population (N = 700)

Variable	All patients (N = 700)
Age, years	67 [56 to 77]
Sex, female	312 (44.6%)
Body mass index, kg/m^2^	26.1 [23.6 to 30]
Admission type	
Operating room	319 (45.6%)
Emergency room	182 (26%)
Ward	111 (15.9%)
Other ICU	41 (5.9%)
External	47 (6.7%)
Hospital discharge disposition	
Ward	526 (75.1%)
Other ICU	134 (19.1%)
Rehabilitation	30 (4.3%)
Home	7 (1%)
Missing	3 (0.4%)
Primary ICU admission diagnosis	
Cardiovascular	200 (28.6%)
Sepsis/infection	108 (15.4%)
Oncologic	98 (14%)
Respiratory	75 (10.7%)
Gastrointestinal	71 (10.1%)
Trauma	59 (8.4%)
Neurologic	49 (7%)
Metabolic/endocrine	29 (4.1%)
Other	11 (1.6%)
Length of ICU stay, days	5 [2 to 10]
Mechanical ventilation	
Received ventilation	473 (67.6%)
Hours (among all patients)	13 [0 to 119]
Hours (among those ventilated)	61 [12 to 205]
Delirium^a^	246 (35.1%)
SAPS II at admission	29 [17 to 40.3]
Marital status (n=695)	
Married/committed partnership	394 (56.7%)
Single	113 (16.3%)
Divorced	89 (12.8%)
Widowed	99 (14.2%)
Return to work at follow-up (n=694)	
Returned to work	108 (15.6%)
Did not return to work	164 (23.6%)
Not applicable or previously retired	422 (60.8%)

n (%) or median [Q1 to Q3]. In case of missing values, the n is indicated in parentheses

ICU, Intensive care unit; SAPS II, Simplified Acute Physiology Score II

^a^Any episode of delirium during the ICU stay

### WHODAS 2.0 and EQ-5D-5L responses

The median EQ-5D-5L index value was 0.81 [IQR 0,52 to 0.94], with highest scores in the mobility [median 2; IQR 1 to 4], life activities [median 2; IQR 1 to 3], and pain [median 2; IQR 1 to 3] domains. The median VAS was 65 [IQR 50 to 80]. The median patient-reported WHODAS 2.0 sum score was 11 [IQR 3 to 23], with highest domain sum scores in the mobility domain [median 4; IQR 1 to 7]. The proxy-reported WHODAS 2.0 sum score was higher [median 16; IQR 6 to 28], also with highest domain sum scores in the mobility domain [median 4; IQR 1 to 7]. Comparing the patient-reported and proxy-reported WHODAS 2.0 responses for patients with both instruments revealed no significant differences (Tables 2, S3).

**Table 2:** Responses to the patient-reported and proxy-reported 12-item WHODAS 2.0 and EQ-5D-5L

Instruments and domains	All patients (N = 700)
Patient-reported WHODAS 2.0	(n = 690)
Sum score, points	11 [3 to 23]
Sum score, % of maximum score	23 [6 to 48]
Domain scores	
Cognition	1 [0 to 3]
Mobility	4 [1 to 7]
Self-care	0 [0 to 4]
Getting along	0 [0 to 2]
Life activities	2 [0 to 5]
Participation	2 [1 to 5]
Proxy-reported WHODAS 2.0	(n = 290)
Sum score	16 [6 to 28]
Sum score, % of maximum score	33 [12.5 to 58]
Domain scores	
Cognition	2 [0 to 4]
Mobility	4 [1 to 7]
Self-care	1 [0 to 4]
Getting along	0.5 [0 to 3]
Life activities	3 [1 to 6]
Participation	3.5 [2 to 6]
EQ-5D-5L	(N = 700)
Index value	0.81 [0.52 to 0.94]
Domain scores	
Mobility	2 [1 to 4]
Self-care	1 [1 to 3]
Life activities	2 [1 to 3]
Pain	2 [1 to 3]
Anxiety/depression	1 [1 to 2]
VAS	65 [50 to 80] (n = 679)

Median [Q1 to Q3]. WHODAS 2.0 sum scores range from 0 to 48 points. WHODAS 2.0 domain scores range from 0 to 8 points

EQ-5D-5L, EuroQol 5-Dimensions 5-Level; VAS, Visual analogue scale; WHODAS 2.0, WHO Disability Assessment Schedule 2.0

We observed ceiling effects for the EQ-5D-5L index value and VAS, with 66% and 33% of responses in the top 20%. Moreover, 13% of patients reached the maximum EQ-5D-5L index value and 3% the maximum VAS. We observed floor effects in the WHODAS 2.0 sum scores, where 45% of responses were in the bottom 20% and 11% reached the minimum score. This suggests limitations of the EQ-5D-5L to capture high HrQoL and limitations of the WHODAS 2.0 to capture low disability.

### Correlations between the EQ-5D-5L, the patient-reported and proxy-reported WHODAS 2.0

**Fig. 1:**
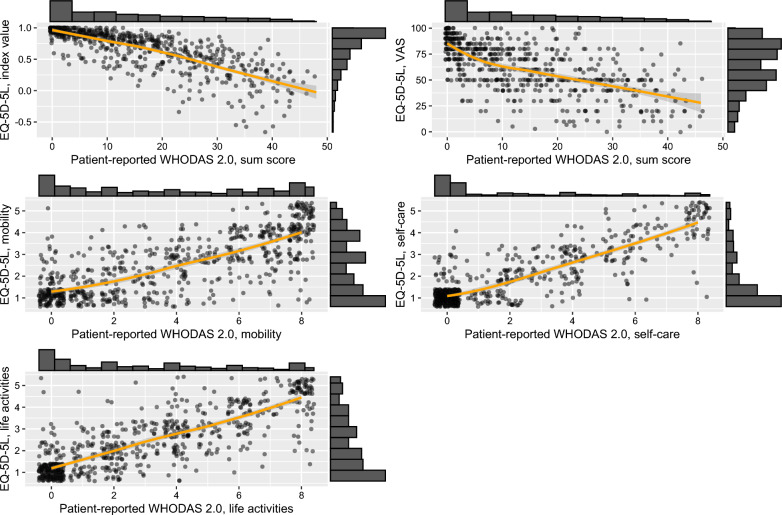
Scatter plots with LOESS curves and histograms comparing the patient-reported WHODAS 2.0 and EQ-5D-5L. WHODAS 2.0 sum scores range from 0 to 48 points. WHODAS 2.0 domain scores range from 0 to 8 points

**Fig. 2:**
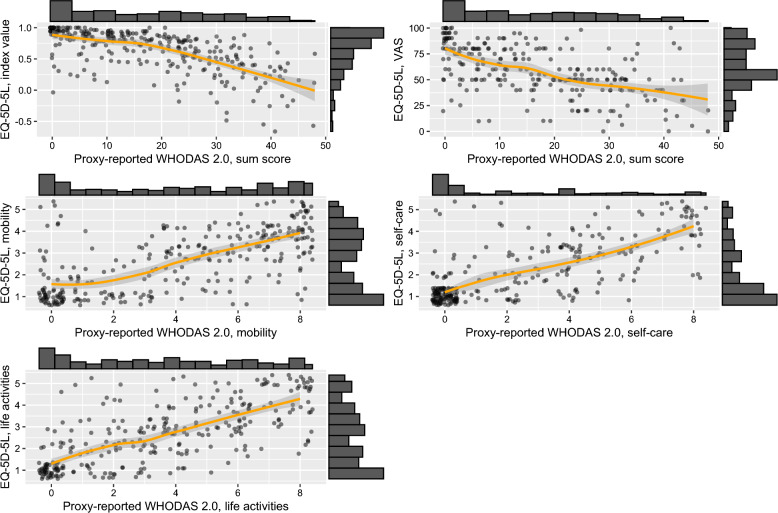
Scatter plots with LOESS curves and histograms comparing the proxy-reported WHODAS 2.0 and EQ-5D-5L. WHODAS 2.0 sum scores range from 0 to 48 points. WHODAS 2.0 domain scores range from 0 to 8 points

Scatter plots and LOESS-curves reveal a consistent linear trend between the EQ-5D-5L index value and the patient-reported WHODAS 2.0 sum score, which was also observed between corresponding EQ-5D-5L and patient-reported WHODAS 2.0 domains. A similar linear trend was found between the EQ-5D-5L index values and the proxy-reported WHODAS 2.0 sum scores as well as corresponding domains. Radar charts depicting EQ-5D-5L responses for different groups of patient-reported and proxy-reported WHODAS 2.0 sum scores revealed that with increasing disability levels, patients indicated greater impairments in the mobility, self-care, and life activities domains of the EQ-5D-5L (Figs. 1, 2, S2, S3).

EQ-5D-5L index values correlated strongly with the patient-reported WHODAS 2.0 sum scores (Spearman: − 0.84 [95% CI − 0.86 to − 0.82]). Corresponding domains of the EQ-5D-5L and the patient-reported WHODAS 2.0 also had strong correlations, namely mobility (0.76 [0.73 to 0.79]), self-care (0.84 [0.82 to 0.86]), and life activities (0.82 [0.80 to 0.85]). The EQ-5D-5L index value also strongly correlated with the proxy-reported WHODAS 2.0 sum score (− 0.70 [− 0.76 to − 0.64]). In the corresponding domains, self-care (0.77 [0.72 to 0.81]) and life activities (0.73 [0.67 to 0.78]) but not mobility (0.67 [0.60 to 0.73]) were strongly correlated. Comparing patient-reported and proxy-reported WHODAS 2.0 revealed strong correlations between sum scores (Spearman: 0.79 [0.74 to 0.83]). We found strong correlations between the mobility (0.78 [0.73 to 0.82]), self-care (0.77 [0.72 to 0.82]), and life activities (0.75 [0.70 to 0.80]) domains, and moderate correlations between the cognition (0.63 [0.56 to 0.70]), getting along (0.56 [0.47 to 0.64]), and participation (0.67 [0.60 to 0.73]) domains (Tables 3, S4).

**Table 3:** Spearman correlation matrix of the patient-reported and proxy-reported 12-item WHODAS 2.0 and the EQ-5D-5L

Instrument and domains	EQ-5D-5L						
	Index value	VAS	Mobility	Self − care	Life activities	Pain	Anxiety/depression
Patient-reported WHODAS 2.0							
Sum score	** − 0.84** [− 0.86 to − 0.82]	− 0.66 [− 0.71 to − 0.62]	**0.74** [0.71 to 0.77]	**0.75** [0.71 to 0.78]	**0.81** [0.78 to 0.83]	0.50 [0.44 to 0.56]	0.53 [0.48 to 0.58]
Mobility	** − 0.76** [− 0.79 to − 0.72]	− 0.56 [− 0.61 to − 0.49]	**0.76** [0.73 to 0.79]	0.67 [0.63 to 0.71]	0.69 [0.65 to 0.73]	0.45 [0.39 to 0.51]	0.36 [0.29 to 0.42]
Self-care	** − 0.71** [− 0.75 to − 0.67]	− 0.52 [− 0.58 to − 0.46]	0.64 [0.60 to 0.68]	**0.84** [0.82 to 0.86]	0.67 [0.63 to 0.71]	0.39 [0.32 to 0.45]	0.39 [0.33 to 0.45]
Cognition	− 0.56 [− 0.61 to − 0.51]	− 0.48 [− 0.54 to − 0.41]	0.43 [0.36 to 0.49]	0.49 [0.43 to 0.55]	0.59 [0.54 to 0.63]	0.34 [0.27 to 0.40]	0.47 [0.40 to 0.52]
Life activities	** − 0.81** [− 0.83 to − 0.78]	− 0.60 [− 0.65 to − 0.55]	**0.70** [0.66 to 0.74]	**0.71** [0.67 to 0.74]	**0.82** [0.80 to 0.85]	0.47 [0.40 to 0.52]	0.50 [0.44 to 0.55]
Getting along	− 0.52 [− 0.57 to − 0.46]	− 0.40 [− 0.46 to − 0.33]	0.41 [0.35 to 0.47]	0.45 [0.38 to 0.51]	0.50 [0.44 to 0.55]	0.32 [0.25 to 0.38]	0.45 [0.39 to 0.51]
Participation	** − 0.71** [− 0.74 to − 0.67]	− 0.62 [− 0.66 to − 0.57]	0.59 [0.45 to 0.64]	0.59 [0.54 to 0.64]	**0.70** [0.66 to 0.74]	0.43 [0.37 to 0.49]	0.55 [0.49 to 0.60]
Proxy-reported WHODAS 2.0							
Sum score	** − 0.70** [− 0.76 to − 0.64]	− 0.57 [− 0.65 to − 0.48]	0.66 [0.59 to 0.72]	0.68 [0.61 to 0.74]	0.69 [0.62 to 0.75]	0.40 [0.30 to 0.49]	0.43 [0.33 to 0.51]
Mobility	− 0.65 [− 0.71 to − 0.58]	− 0.54 [− 0.62 to − 0.44]	0.67 [0.60 to 0.73]	0.57 [0.49 to 0.65]	0.60 [0.52 to 0.67]	0.39 [0.29 to 0.49]	0.35 [0.24 to 0.45]
Self-care	− 0.66 [− 0.72 to − 0.58]	− 0.50 [− 0.59 to − 0.40]	0.58 [0.50 to 0.65]	**0.77** [0.72 to 0.81]	0.64 [0.56 to 0.70]	0.37 [0.26 to 0.46]	0.34 [0.23 to 0.43]
Cognition	− 0.59 [− 0.66 to − 0.51]	− 0.45 [− 0.55 to − 0.35]	0.53 [0.44 to 0.61]	0.56 [0.48 to 0.64]	0.58 [0.50 to 0.66]	0.32 [0.21 to 0.42]	0.37 [0.26 to 0.46]
Life activities	** − 0.71** [− 0.76 to − 0.65]	− 0.58 [− 0.65 to − 0.48]	0.66 [0.59 to 0.72]	0.69 [0.62 to 0.75]	**0.73** [0.67 to 0.78]	0.39 [0.29 to 0.48]	0.43 [0.33 to 0.52]
Getting along	− 0.50 [− 0.58 to − 0.41]	− 0.36 [− 0.47 to − 0.25]	0.44 [0.34 to 0.53]	0.49 [0.40 to 0.58]	0.52 [0.43 to 0.60]	0.26 [0.15 to 0.37]	0.36 [0.25 to 0.46]
Participation	− 0.64 [− 0.70 to − 0.56]	− 0.52 [− 0.61 to − 0.42]	0.61 [0.53 to 0.67]	0.57 [0.48 to 0.64]	0.63 [0.55 to 0.69]	0.38 [0.28 to 0.48]	0.41 [0.31 to 0.51]
EQ-5D-5L							
VAS	0.67 [0.62 to 0.71]						

Coefficient [95% CI]. *p* < 0.05 for all Spearman correlation coefficients. A coefficient of 0.1 to 0.39 was considered weak, 0.40 to 0.69 was considered moderate, 0.70 to 0.89 was considered strong, and ≥ 0.90 was considered very strong. Strong correlations (Spearman ≥ 0.70) are highlighted in bold. WHODAS 2.0 sum scores range from 0 to 48 points. WHODAS 2.0 domain scores range from 0 to 8 points

EQ-5D-5L, EuroQol 5-Dimensions 5-Level; VAS, Visual analogue scale; WHODAS 2.0, WHO Disability Assessment Schedule 2.0

### Univariable and multivariable linear regressions

Univariable linear regressions revealed a negative association between patient-reported WHODAS 2.0 sum scores and EQ-5D-5L index values (− 0.02 [95% CI − 0.02 to – 0.02]; *p* < 0.01) and the EQ-5D-5L VAS (− 1.22 [− 1.34 to − 1.11]; *p* < 0.01). We also found significant associations between corresponding domains of the EQ-5D-5L and the patient-reported WHODAS 2.0, namely mobility (0.34 [0.32 to 0.36]; *p* < 0.01), self-care (0.41 [0.39 to 0.43]; *p* < 0.01), and life activities (0.4 [0.38 to 0.42];* p* < 0.01). Adjusted R^2^ ranged from 0.4 to 0.77. After adjusting for additional covariates, the patient-reported WHODAS 2.0 and its domains were still significantly associated with the EQ-5D-5L index value, VAS and corresponding domains, with coefficients and goodness of fit similar to the univariable regressions (Tables 4, S5).

**Table 4:** Multivariable regression models predicting the EQ-5D-5L with the patient-reported WHODAS 2.0 and additional covariables

Independent variables	Dependent variables				
	EQ-5D-5L index value	EQ-5D-5L VAS	EQ-5D-5L mobility	EQ-5D-5L self-care	EQ-5D-5L life activities
Patient-reported WHODAS 2.0, sum score	− 0.02*** (− **0.02 to − 0.02)**	− 1.22*** (− **1.35 to − 1.10)**			
Mobility			0.34*** (0.31 to 0.36)		
Self-care				0.41*** (0.39 to 0.42)	
Life activities					0.39*** (0.37 to 0.42)
Patient sex, male	− 0.001 (− 0.03 to 0.03)	− 1.63 (− 4.59 to 1.34)	0.14** (0.01 to 0.27)	0.08* (− 0.01 to 0.17)	0.08 (− 0.04 to 0.19)
Patient age, years	− 0.0002 (− 0.001 to 0.001)	− 0.06 (− 0.16 to 0.04)	0.0005 (− 0.004 to 0.005)	0.002 (− 0.001 to 0.005)	− 0.001 (− 0.004 to 0.003)
ICU Length of stay, days	0.003** (0.0002 to 0.01)	− 0.3* (− 0.62 to 0.02)	− 0.001 (− 0.01 to 0.005)	0.001 (− 0.002 to 0.003)	0.01** (0.01 to 0.02)
Mechanical ventilation, hours	− 0.0001* (− 0.003 to 0.0)	0.01* (− 0.001 to 0.03)	0.0 (− 0.001 to 0.01)	0.0004* (− 0.0 to 0.001)	0.0 (− 0.001 to 0.0005)
SAPS II score at admission	0.0004 (− 0.001 to 0.001)	0.002 (− 0.09 to 0.10)	0.003 (− 0.001 to 0.01)	0.001 (− 0.002 to 0.004)	0.001 (− 0.003 to 0.005)
Delirium, yes/no	− 0.003 (− 0.04 to 0.03)	− 0.93 (− 4.51 to 2.66)	− 0.06 (− 0.21 to 0.09)	− 0.05 (− 0.15 to 0.06)	− 0.02 (− 0.16 to 0.12)
Constant	0.98*** (0.89 to 1.06)	85.94*** (77.52 to 94.36)	1.04*** (0.68 to 1.39)	0.92*** (0.68 to 1.16)	1.12*** (0.8 to 1.44)
N	687	600	687	692	683
R^2^	0.63	0.41	0.60	0.77	0.68
Adjusted R^2^	0.63	0.40	0.60	0.77	0.68
Residual standard error	0.19 (df = 679)	18.11 (df = 592)	0.85 (df = 679)	0.58 (df = 684)	0.76 (df = 675)
F statistic	167.11*** (df = 7; 679)	58.96*** (df = 7; 592)	145.83*** (df = 7; 679)	333.35*** (df = 7; 684)	207.64*** (df = 7; 675)

Significant coefficients (*p* < 0.05) are highlighted in bold. Coefficients are displayed with 95% CIs. WHODAS 2.0 sum scores range from 0 to 48 points. WHODAS 2.0 domain scores range from 0 to 8 points

EQ-5D-5L, EuroQol 5-Dimensions 5-Level; ICU, Intensive care unit; VAS, Visual analogue scale; WHODAS 2.0, WHO Disability Assessment Schedule 2.0

*p < 0.1; **p < 0.05; ***p < 0.01

For the proxy-reported WHODAS 2.0, univariable linear regressions revealed a negative association between WHODAS 2.0 sum scores and EQ-5D-5L index values (− 0.02 [95% CI − 0.02 to − 0.01]; *p* < 0.01) and EQ-5D-5L VAS (− 1.03 [− 1.22 to − 0.83]; *p* < 0.01). The EQ-5D-5L and proxy-reported WHODAS 2.0 domains mobility (0.31 [0.27 to 0.35]; *p* < 0.01), self-care (0.36 [0.32 to 0.40]; *p* < 0.01), and life activities (0.37 [0.32 to 0.41]; *p* < 0.01) were significantly associated. Adjusted R^2^ ranged from 0.3 to 0.55. Multivariable linear regressions with additional covariables yielded significant relationships of similar range but had better fit (adjusted R^2^ ranging from 0.32 to 0.57). Overall, regressions predicting the EQ-5D-5L with the proxy-reported WHODAS 2.0 yielded lower R^2^ than regressions predicting the EQ-5D-5L with the patient-reported WHODAS 2.0 (Tables 5, S6).

**Table 5:** Multivariable regression models predicting the EQ-5D-5L with the proxy-reported WHODAS 2.0 and additional covariables

Independent variables	Dependent variables				
	EQ-5D-5L index value	EQ-5D-5L VAS	EQ-5D-5L mobility	EQ-5D-5L self-care	EQ-5D-5L life activities
Proxy-reported WHODAS 2.0, sum score	− 0.02*** (− **0.02 to − 0.02)**	− 1.04*** (− **1.24 to − 0.84)**			
Mobility			0.31*** (0.27 to 0.35)		
Self-care				0.35*** (0.32 to 0.39)	
Life activities					0.37*** (0.33 to 0.41)
Patient sex, male	− 0.001 (− 0.06 to 0.06)	− 0.87 (− 5.94 to 4.2)	0.06 (− 0.18 to 0.31)	0.2* (− 0.01 to 0.41)	− 0.04 (− 0.27 to 0.19)
Patient age, years	− 0.001 (− 0.003 to 0.001)	− 0.24*** (− **0.41 to − 0.08)**	− 0.002 (− 0.01 to 0.01)	0.01** (0.0003 to 0.01)	0.002 (− 0.01 to 0.01)
ICU Length of stay, days	0.01*** (0.005 to 0.02)	0.08 (− 0.042 to 0.58)	− 0.02 (− 0.04 to 0.001)	− 0.02* (− 0.04 to 0.001)	− 0.02 (− 0.04 to 0.01)
Mechanical ventilation, hours	– 0.0004*** (− **0.001 to − 0.0001)**	0.01(− 0.02 to 0.03)	0.001 (− 0.001 to 0.002)	0.001 (− 0.0002 to 0.002)	0.0004 (− 0.001 to 0.001)
SAPS II score at admission	0.001 (− 0.001 to 0.003)	− 0.04 (− 0.2 to 0.12)	0.004 (− 0.003 to 0.01)	0.001 (− 0.01 to 0.01)	− 0.002 (− 0.01 to 0.01)
Delirium, yes/no	0.02 (− 0.05 to 0.09)	2.07 (− 3.94 to 8.07)	− 0.23 (− 0.51 to 0.06)	− 0.29** (− **0.54 to − 0.05)**	− 0.23 (− 0.51 to 0.05)
Constant	0.93*** (0.77 to 1.09)	91.28*** (77.31 to 105.24)	1.61*** (0.97 to 2.25)	0.97*** (0.42 to 1.52)	1.54*** (0.93 to 2.16)
N	289	246	284	291	283
R^2^	0.46	0.34	0.47	0.58	0.52
Adjusted R^2^	0.45	0.32	0.45	0.57	0.51
Residual standard error	0.25 (df = 281)	19.77 (df = 238)	1.01 (df = 276)	0.88 (df = 283)	0.97 (df = 275)
F statistic	34.46*** (df = 7; 281)	17.79*** (df = 7; 238)	34.47*** (df = 7; 276)	55.11*** (df = 7; 283)	43.2*** (df = 7; 275)

Significant coefficients (*p* < 0.05) are highlighted in bold. Coefficients are displayed with 95% CIs. WHODAS 2.0 sum scores range from 0 to 48 points. WHODAS 2.0 domain scores range from 0 to 8 points

EQ-5D-5L, EuroQol 5-Dimensions 5-Level; ICU, Intensive care unit; VAS, Visual analogue scale; WHODAS 2.0, WHO Disability Assessment Schedule 2.0

*p < 0.1; **p < 0.05; ***p < 0.01

## Discussion

### Key findings

This study demonstrates a strong correlation between the patient-reported WHODAS 2.0, the proxy-reported WHODAS 2.0, and the EQ-5D-5L index value among ICU survivors. We also found strong correlations between corresponding domains of the instruments, namely mobility, self-care, and life activities. While patient-reported and proxy-reported versions of the WHODAS 2.0 were also closely correlated, the patient-reported WHODAS 2.0 showed stronger correlations with the EQ-5D-5L than the proxy-reported WHODAS 2.0. For all instruments, we found substantial ceiling and floor effects, indicating limitations of the WHODAS 2.0 and EQ-5D-5L to capture low levels of disability and high HrQoL.

### What is already known

The patient-reported WHODAS 2.0 sum score of 11 points (equaling 23%) and the proxy-reported WHODAS 2.0 sum score of 16 points (equaling 33%) indicate substantial disability among our post-ICU cohort. Those median WHODAS 2.0 sum scores correspond to the 90-95th percentile of a representative population sample of 8,841 Australian adults [31]. Our patient-reported WHODAS 2.0 sum scores align with previous studies among ICU survivors, which found median patient-reported WHODAS 2.0 scores ranging from 17% (equivalent to approximately 8 points) to 23% of the maximum score (equivalent to approximately 11 points) [22, 32]. Another study of 262 ICU survivors found that 50% reported mild disability, corresponding to patient-reported WHODAS 2.0 sum scores between 2 and 12 points [33].

Our median EQ-5D-5L index score of 0.81 and median EQ-5D-5L VAS of 65 were below the German population norms of 0.88 and 71.59, respectively [34]. However, our findings align with a Portuguese cohort study among 275 ICU survivors, which found an EQ-5D-5L index value of 0.81 6 months after ICU discharge [35], and a German retrospective cohort study of 217 ICU survivors, which found an EQ-5D-5L index value of 0.8 after 1 year [36]. Another Australian study reported higher EQ-5D-5L index values among ICU survivors (median of 0.92 after 6 months), which may be due to the exclusion of patients hospitalized at follow ups [23]. Similar to our study, that study also found strong Spearman correlation coefficients between the patient-reported WHODAS 2.0 sum scores and EQ-5D-5L index values (− 0.81) as well as VAS (− 0.72) [23]. A domain-specific analysis, however, was not conducted. Similarly, an Australian prospective cohort study among ICU survivors found that patients with moderate or severe disability in the patient-reported WHODAS 2.0 showed worse EQ-5D-5L index values [33]. The study found greatest HrQoL reductions in the domains of mobility, self-care, and life activities, which are the overlapping domains of both instruments [33]. The authors interpreted this as a causal link between the concepts of disability and HrQoL, suggesting that higher levels of disability lead to lower quality of life [33].

We are the first to assess the correlation between patient-reported and proxy-reported WHODAS 2.0 in a post-ICU population. The observed strong correlations are consistent with a recent study on patients with traumatic brain injury (Spearman: 0.74) [37]. Yet, further research is needed to determine whether a proxy can reliably approximate the patient’s perspective among ICU survivors.

The significant floor and ceiling effects of the WHODAS 2.0 sum score and the EQ-5D-5L index value highlight limitations in the ability to assess low levels of disability and high levels of HrQoL. In contrast to our findings, previous studies did not observe relevant floor effects for the patient-reported WHODAS 2.0 after 6 months [22] or ceiling effects for the EQ-5D-5L VAS [23], which were defined as ≥ 15% of participants having the lowest or highest possible value. However, a relevant ceiling effect was observed for the EQ-5D-5L index value after 6 months, with 22.2% of participants having the highest value [23].

### What this study adds and practical implications

The strong correlations between the WHODAS 2.0 and the EQ-5D-5L may indicate a connection between the concepts of disability and HrQoL. While the WHO has argued that disability describes what a person “does” and HrQoL captures what a person “feels” [19], a more detailed comparison of the WHODAS 2.0 and EQ-5D-5L reveals substantial overlap. Both instruments share key domains—mobility, self-care, and life activities—and include nearly identical items. One reason for the similarity of the instruments lies in how their domains were selected: both the EuroQol group and the WHO analyzed existing instruments to identify commonly used domains [16, 19, 38]. However, previous research has often treated HrQoL and disability as separate concepts, implying a causal relationship between both [33, 39–41]. Treating disability and HrQoL as distinct entities comes with the risk of misinterpreting their correlation as causality. Instead, the instruments may measure fundamentally similar aspects of health.

The substantial overlap between the WHODAS 2.0 and EQ-5D-5L domains causes redundancy and collinearity in post-ICU assessments. Using both instruments may also increase patient burden of follow-up assessments, which may curb dropout rates and compromise the accuracy of subsequent tests due to fatigue. Selecting one instrument aligned with the assessment's primary goals, which could be either the WHODAS 2.0 or the EQ-5D-5L, supplemented by non-overlapping tools as needed, could streamline assessments, reduce patient strain, and improve resource efficiency. The EQ-5D-5L offers the advantage of quality-adjusted life year calculation, provides an assessment of pain and depression, and has fewer items than the WHODAS 2.0. However, it does not explicitly include the domains cognition, communication, and participation, which are covered by the WHODAS 2.0. Our regression analysis may also be used to derive an approximation of the EQ-5D-5L with existing WHODAS 2.0 results. Both instruments have proxy versions available, potentially reducing selection bias in cohorts with greater impairments. However, to our knowledge, the proxy version of EQ-5D-5L has not yet been used among survivors of critical illness.

### Strengths and limitations

This study’s strengths include the multimodal follow-up, including home visits, which reduced the selection bias and allowed the follow-up of patients with high dependency levels. Additionally, due to its broad inclusion criteria, a diverse cohort of patients from surgical, medical, and mixed ICUs was included in the analysis. However, our study is subject to limitations. Firstly, ERIC was not powered to analyze the relationship between the WHODAS 2.0 and the EQ-5D-5L. Secondly, the study's geographic focus on the metropolitan area of Berlin, Germany, could have introduced a selection bias and limits the generalizability of our findings to other populations and healthcare systems. Thirdly, the observed correlations could partially be due to chance factors. Hence, prospective studies should verify our results and may also further analyze the transferability of scores between the WHODAS 2.0 and EQ-5D-5L.

## Conclusion

In this secondary analysis of a large cluster-randomized controlled ICU trial, we found that patient-reported and proxy-reported WHODAS 2.0 sum scores are strongly linked to the EQ-5D-5L index values. Additionally, corresponding domains of both instruments, namely mobility, self-care, and life activities, are strongly correlated. The patient-reported WHODAS 2.0 showed stronger correlations with the EQ-5D-5L than the proxy-reported WHODAS 2.0. Further, we found substantial ceiling and floor effects for both instruments. Considering the overlap of both instruments, including some nearly identical questions, the concurrent use of the WHODAS 2.0 and the EQ-5D-5L may not be meaningful as it could introduce redundancy and increase patient burden of ICU follow-ups. Thus, we recommend selecting either the WHODAS 2.0 or the EQ-5D-5L for post-ICU follow-ups.

## Supplementary Information

Additional file 1

Additional file 2

## Data Availability

The datasets used and/or analyzed during the current study are available from the corresponding author upon reasonable scientific and non-commercial request.

## Abbreviations

CI: Confidence interval
EQ-5D-5L: EuroQol 5-Dimensions 5-Level
ERIC: Enhanced Recovery after Intensive Care
HrQoL: Health-related quality of life
ICU: Intensive care unit
IQR: Interquartile range
LOESS: Locally estimated scatterplot smoothing
PICS: Post-intensive care syndrome
SAPS II: Simplified Acute Physiology Score II
VAS: Visual analogue scale
WHO: World Health Organization
WHODAS 2.0: World Health Organization Disability Assessment Schedule 2.0
