# Supplementary material for: Health-related quality of life correlates with patient-reported and proxy-reported disability in critical illness survivors: a secondary analysis of the ERIC trial
Source: Crit Care. 2025 Apr 23;29:158. doi: 10.1186/s13054-025-05399-3 (PMC12020060; doi:10.1186/s13054-025-05399-3)
Supplement: Supplementary file 1 — Additional file 1 [file 13054_2025_5399_MOESM1_ESM.pdf]

**Table S1.** Instruments that were used at the three-month and six-month follow-up assessments.

| Domain | Instrument | Abbreviation | Follow-up 1 (after 3 months) | Follow-up 2 (after 6 months) |
|---|---|---|---|---|
| *Cognition* | MiniCog | - | ✓ | ✓ |
| | Animal Naming Test | ANT | ✓ | ✓ |
| | Trail Making Test A and B | TMT A/B | | ✓ |
| | Repeatable Battery for the Assessment of Neuropsychological Status | RBANS | | ✓ |
| *Mental health* | Patient Health Questionnaire 4 | PHQ-4 [a] | ✓ | |
| | Patient Health Questionnaire 8 | PHQ-8 | | ✓ |
| | Generalized Anxiety Disorder Scale 7 | GAD-7 | | ✓ |
| | Impact of Event Scale – revised | IES-R | | ✓ |
| *Physical function* | Timed Up-and-Go | TUG | ✓ | ✓ |
| | Handgrip strength | HGS | ✓ | ✓ |
| | 2-Minute Walk Test | 2-MWT | | ✓ |
| | Short Physical Performance Battery | SPPB | | ✓ |
| *Disability* | 12-item WHO Disability Assessment Schedule 2.0 (patient-reported and proxy-reported) | WHODAS 2.0 | | ✓ |
| *Health-related quality of life* | EuroQol 5-Dimensions 5-Level | EQ-5D-5L | ✓ | ✓ |
| *Subjective health* | Items on subjective mental and physical health before and after the ICU and specific health concerns | - | ✓ | ✓ |

[a] Including the subscales Patient Health Questionnaire 2 (PHQ-2) and Generalized Anxiety Disorder Scale 2 (GAD-2).

The instruments used at the follow-ups are described in Spies CD, Krampe H, Paul N, et al: Instruments to measure outcomes of post-intensive care syndrome in outpatient care settings – results of an expert consensus and feasibility field test. J Intensive Care Soc 2021; 22(2):159-174. doi:10.1177/1751143720923597. ICU, intensive care unit.

**Table S2.** Baseline characteristics of patients with a completed patient-reported and proxy-reported WHODAS 2.0.

| Variable | Patients with completed patient-reported WHODAS 2.0 (n=690) | Patients with completed proxy-reported WHODAS 2.0 (n=290) |
|---|---|---|
| Age, years | 67 [56 to 76] | 69 [57.3 to 78] |
| Sex, female | 310 (44.9%) | 125 (43.1%) |
| Body mass index, kg/m² | 26.1 [23.6 to 30] | 26.1 [23.4 to 29.7] |
| Admission type | | |
| Operating room | 318 (46.1%) | 124 (42.8%) |
| Emergency room | 179 (25.9%) | 75 (25.9%) |
| Ward | 107 (15.5%) | 50 (17.2%) |
| Other ICU | 41 (5.9%) | 15 (5.2%) |
| External | 45 (6.5%) | 26 (9%) |
| Hospital discharge disposition | | |
| Ward | 519 (75.2%) | 209 (72.1%) |
| Other ICU | 132 (19.1%) | 60 (20.7%) |
| Rehabilitation | 29 (4.2%) | 17 (5.9%) |
| Home | 7 (1%) | 4 (1.4%) |
| Missing | 3 (0.4%) | 0 (0%) |
| Primary ICU admission diagnosis | | |
| Cardiovascular | 197 (28.6%) | 81 (27.9%) |
| Sepsis/infection | 107 (15.5%) | 51 (17.6%) |
| Oncologic | 97 (14.1%) | 44 (15.2%) |
| Respiratory | 75 (10.9%) | 35 (12.1%) |
| Gastrointestinal | 69 (10%) | 25 (8.6%) |
| Trauma | 58 (8.4%) | 22 (7.6%) |
| Neurologic | 48 (7%) | 21 (7.2%) |
| Metabolic/endocrine | 29 (4.2%) | 8 (2.8%) |
| Other | 10 (1.4%) | 3 (1%) |
| Length of ICU stay, days | 4 [2 to 10] | 5 [3 to 11] |
| Mechanical ventilation | | |
| Received ventilation | 464 (67.2%) | 211 (72.8%) |
| Hours (among all patients) | 12 [0 to 116] | 20 [0 to 166] |
| Hours (among those ventilated) | 56.5 [11 to 202] | 71 [9.5 to 262] |
| Delirium [a] | 240 (34.8%) | 112 (38.6%) |
| SAPS II at admission | 28 [17 to 40] | 32 [19.3 to 44] |
| Marital status | (n=686) | (n=286) |
| Married/committed partnership | 387 (56.4%) | 200 (69.9%) |
| Single | 113 (16.5%) | 35 (12.2%) |
| Divorced | 89 (13%) | 25 (8.7%) |
| Widowed | 97 (14.1%) | 26 (9.1%) |
| Return to work at follow-up | (n=686) | (n=287) |
| Returned to work | 107 (15.6%) | 34 (11.8%) |
| Did not return to work | 162 (23.6%) | 70 (24.4%) |
| Not applicable or previously retired | 417 (60.8%) | 183 (63.8%) |

n (%) or median [Q1 to Q3]. In case of missing values, the n is indicated in parentheses. [a] Any episode of delirium during ICU stay. ICU, intensive care unit; SAPS II, Simplified Acute Physiology Score II.

**Table S3.** Comparison of patient-reported and proxy-reported WHODAS 2.0.

| | **Patient-reported WHODAS 2.0 (n=280)** | **Proxy-reported WHODAS 2.0 (n=280)** | ***p*** |
|---|---|---|---|
| Sum score, points | 14 [5.75 to 27.3] | 16 [5.75 to 27] | 0.144 |
| Sum score, % of maximum score | 29 [12 to 57] | 33 [12.5 to 56] | 0.126 |
| Domain scores | | | |
| Cognition | 2 [0 to 4] | 2 [0 to 4] | 0.08 |
| Mobility | 4 [1 to 7] | 4 [1 to 7] | 0.375 |
| Self-care | 1 [0 to 4] | 1 [0 to 4] | 0.793 |
| Getting along | 0 [0 to 3] | 0 [0 to 3] | 0.26 |
| Life activities | 3 [0 to 6] | 3 [1 to 6] | 0.92 |
| Participation | 3 [1 to 5] | 3 [2 to 6] | 0.192 |

Median [Q1 to Q3]. WHODAS 2.0 sum scores range from 0 to 48 points. WHODAS 2.0 domain scores range from 0 to 8 points. Statistical comparisons between patient-reported and proxy-reported scores were made using the Wilcoxon signed-rank test. WHODAS 2.0, WHO Disability Assessment Schedule 2.0.

**Table S4.** Spearman correlation coefficients between patient-reported and proxy-reported WHODAS 2.0.

| **Proxy-reported WHODAS 2.0** | **Patient-reported WHODAS 2.0** | | | | | | |
|---|---|---|---|---|---|---|---|
| | Sum score | Cognition | Mobility | Self-care | Getting along | Life activities | Participation |
| Sum score | **0.79 [0.74 to 0.83]** | 0.61 [0.53 to 0.68] | **0.71 [0.65 to 0.76]** | 0.69 [0.63 to 0.75] | 0.48 [0.39 to 0.57] | **0.72 [0.66 to 0.77]** | 0.66 [0.59 to 0.73] |
| Cognition | 0.66 [0.59 to 0.73] | 0.63 [0.56 to 0.70] | 0.52 [0.43 to 0.60] | 0.57 [0.49 to 0.65] | 0.48 [0.38 to 0.57] | 0.60 [0.52 to 0.67] | 0.55 [0.46 to 0.62] |
| Mobility | **0.70 [0.63 to 0.75]** | 0.47 [0.37 to 0.56] | **0.78 [0.73 to 0.82]** | 0.59 [0.51 to 0.66] | 0.31 [0.20 to 0.41] | 0.64 [0.56 to 0.70] | 0.55 [0.46 to 0.63] |
| Self-care | **0.73 [0.67 to 0.79]** | 0.54 [0.45 to 0.62] | 0.65 [0.57 to 0.71] | **0.77 [0.72 to 0.82]** | 0.44 [0.34 to 0.53] | 0.69 [0.63 to 0.75] | 0.60 [0.51 to 0.67] |
| Getting along | 0.60 [0.52 to 0.67] | 0.53 [0.44 to 0.61] | 0.43 [0.33 to 0.52] | 0.49 [0.40 to 0.58] | 0.56 [0.47 to 0.64] | 0.51 [0.42 to 0.60] | 0.53 [0.44 to 0.61] |
| Life activities | **0.79 [0.74 to 0.83]** | 0.61 [0.53 to 0.68] | **0.71 [0.64 to 0.76]** | **0.70 [0.64 to 0.76]** | 0.44 [0.34 to 0.53] | **0.75 [0.70 to 0.80]** | 0.64 [0.57 to 0.71] |
| Participation | 0.69 [0.63 to 0.75] | 0.53 [0.43 to 0.61] | 0.57 [0.48 to 0.64] | 0.59 [0.51 to 0.66] | 0.45 [0.35 to 0.53] | 0.64 [0.56 to 0.70] | 0.67 [0.60 to 0.73] |

Coefficient [95% CI]. $p<0.05$ for all Spearman correlation coefficients. A coefficient of 0.1 to 0.39 was considered weak, 0.40 to 0.69 was considered moderate, 0.7 to 0.89 was considered strong, and ≥0.90 was considered very strong. Strong correlations (Spearman ≥0.7) are highlighted in bold. WHODAS 2.0 sum scores range from 0 to 48 points. WHODAS 2.0 domain scores range from 0 to 8 points. WHODAS 2.0, WHO Disability Assessment Schedule 2.0.

**Table S5.** Univariable regression models predicting the EQ-5D-5L with the patient-reported WHODAS 2.0.

| **Independent variables** | **Dependent variables** | | | | |
|---|---|---|---|---|---|
| | **EQ-5D-5L index value** | **EQ-5D-5L VAS** | **EQ-5D-5L mobility** | **EQ-5D-5L self-care** | **EQ-5D-5L life activities** |
| Patient-reported WHODAS 2.0, sum score | **-0.02$^{***}$ (-0.02 to -0.02)** | **-1.22$^{***}$ (-1.34 to -1.11)** | | | |
| Mobility | | | **0.34$^{***}$ (0.32 to 0.36)** | | |
| Self-care | | | | **0.41$^{***}$ (0.39 to 0.43)** | |
| Life activities | | | | | **0.4$^{***}$ (0.38 to 0.42)** |
| Constant | **0.98$^{***}$ (0.96 to 1.01)** | **79.57$^{***}$ (77.39 to 81.75)** | **1.19$^{***}$ (1.09 to 1.29)** | **1.06$^{***}$ (1.01 to 1.11)** | **1.19$^{***}$ (1.1 to 1.27)** |
| N | 690 | 603 | 690 | 695 | 686 |
| $R^2$ | 0.63 | 0.41 | 0.6 | 0.77 | 0.68 |
| Adjusted $R^2$ | 0.63 | 0.40 | 0.6 | 0.77 | 0.68 |
| Residual standard error | 0.19 (df = 688) | 18.07 (df = 601) | 0.85 (df = 688) | 0.59 (df = 693) | 0.76 (df = 684) |
| F statistic | 1,159.57$^{***}$ (df = 1; 688) | 410.44$^{***}$ (df = 1; 601) | 1,018.84$^{***}$ (df = 1; 688) | 2,292.63$^{***}$ (df = 1; 693) | 1,441.54$^{***}$ (df = 1; 684) |

$^{*}p<0.1$; $^{**}p<0.05$; $^{***}p<0.01$. Significant coefficients ($p<0.05$) are highlighted in bold. Coefficients are displayed with 95% CIs. WHODAS 2.0 sum scores range from 0 to 48 points. WHODAS 2.0 domain scores range from 0 to 8 points. EQ-5D-5L, EuroQol 5-Dimensions 5-Level; ICU, intensive care unit; VAS, visual analog scale; WHODAS 2.0, WHO Disability Assessment Schedule 2.0.

**Table S6.** Univariable regression models predicting the EQ-5D-5L with the proxy-reported WHODAS 2.0.

| Independent variables | Dependent variables | | | | |
|---|---|---|---|---|---|
| | **EQ-5D-5L index value** | **EQ-5D-5L VAS** | **EQ-5D-5L mobility** | **EQ-5D-5L self-care** | **EQ-5D-5L life activities** |
| Proxy-reported WHODAS 2.0, sum score | **-0.02$^{***}$ (-0.02 to -0.01)** | **-1.03$^{***}$ (-1.22 to -0.83)** | | | |
| Mobility | | | **0.31$^{***}$ (0.27 to 0.35)** | | |
| Self-care | | | | **0.36$^{***}$ (0.32 to 0.40)** | |
| Life activities | | | | | **0.37$^{***}$ (0.32 to 0.41)** |
| Constant | **0.94$^{***}$ (0.89 to 0.99)** | **76.05$^{***}$ (71.93 to 80.16)** | **1.39$^{***}$ (1.2 to 1.59)** | **1.21$^{***}$ (1.07 to 1.34)** | **1.36$^{***}$ (1.17 to 1.54)** |
| N | 290 | 247 | 285 | 292 | 284 |
| $R^2$ | 0.43 | 0.31 | 0.45 | 0.55 | 0.51 |
| Adjusted $R^2$ | 0.43 | 0.30 | 0.45 | 0.55 | 0.51 |
| Residual standard error | 0.25 (df = 288) | 20.05 (df = 245) | 1.02 (df = 283) | 0.90 (df = 290) | 0.97 (df = 282) |
| F statistic | 218.62$^{***}$ (df = 1; 288) | 108.17$^{***}$ (df = 1; 245) | 234.09$^{***}$ (df = 1; 283) | 360.43$^{***}$ (df = 1; 290) | 297.72$^{***}$ (df = 1; 282) |

$^{*}p<0.1$; $^{**}p<0.05$; $^{***}p<0.01$. Significant coefficients ($p<0.05$) are highlighted in bold. Coefficients are displayed with 95% CIs. WHODAS 2.0 sum scores range from 0 to 48 points. WHODAS 2.0 domain scores range from 0 to 8 points. EQ-5D-5L, EuroQol 5-Dimensions 5-Level; ICU, intensive care unit; VAS, visual analog scale; WHODAS 2.0, WHO Disability Assessment Schedule 2.0.

**Figure S1.** Flowchart of patients included in the analysis.

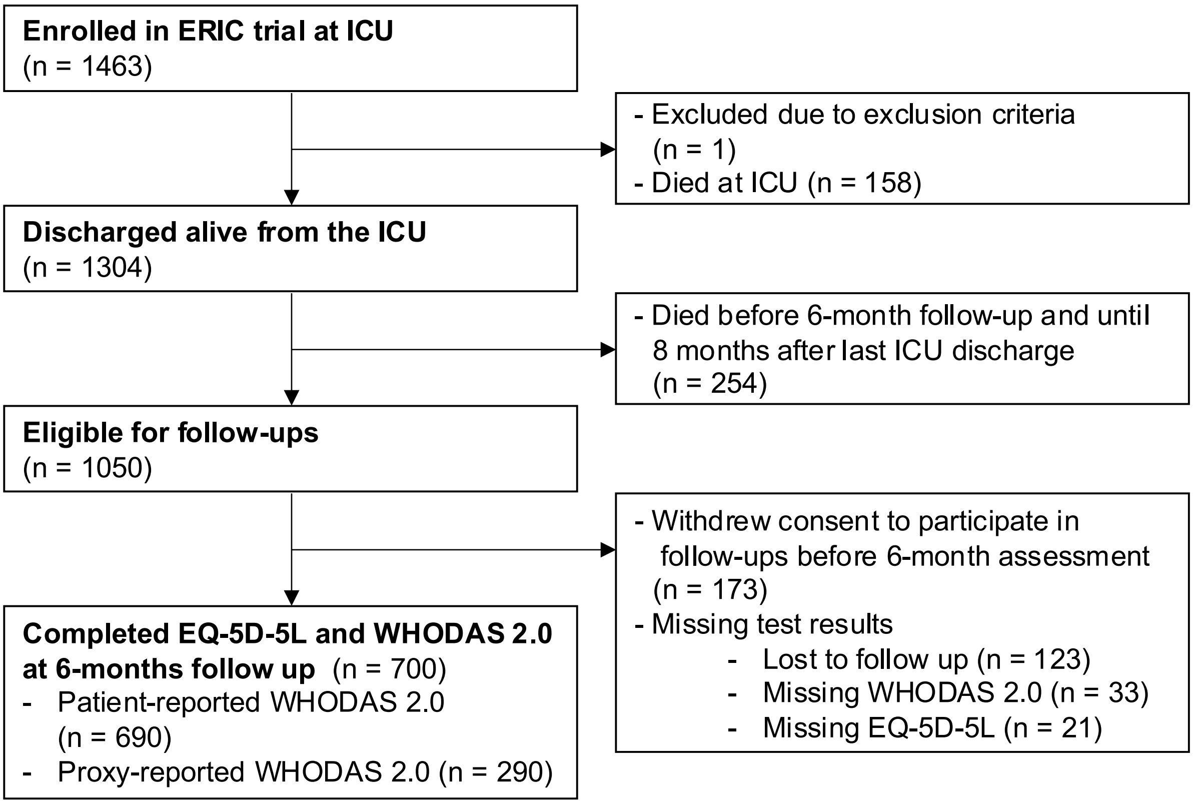


ICU, intensive care unit; EQ-5D-5L, EuroQol 5-Dimensions 5-Level; WHODAS 2.0, WHO Disability Assessment Schedule 2.0.

**Figure S2.** EQ-5D-5L domain results, stratified by patient-reported WHODAS 2.0 sum score groups.

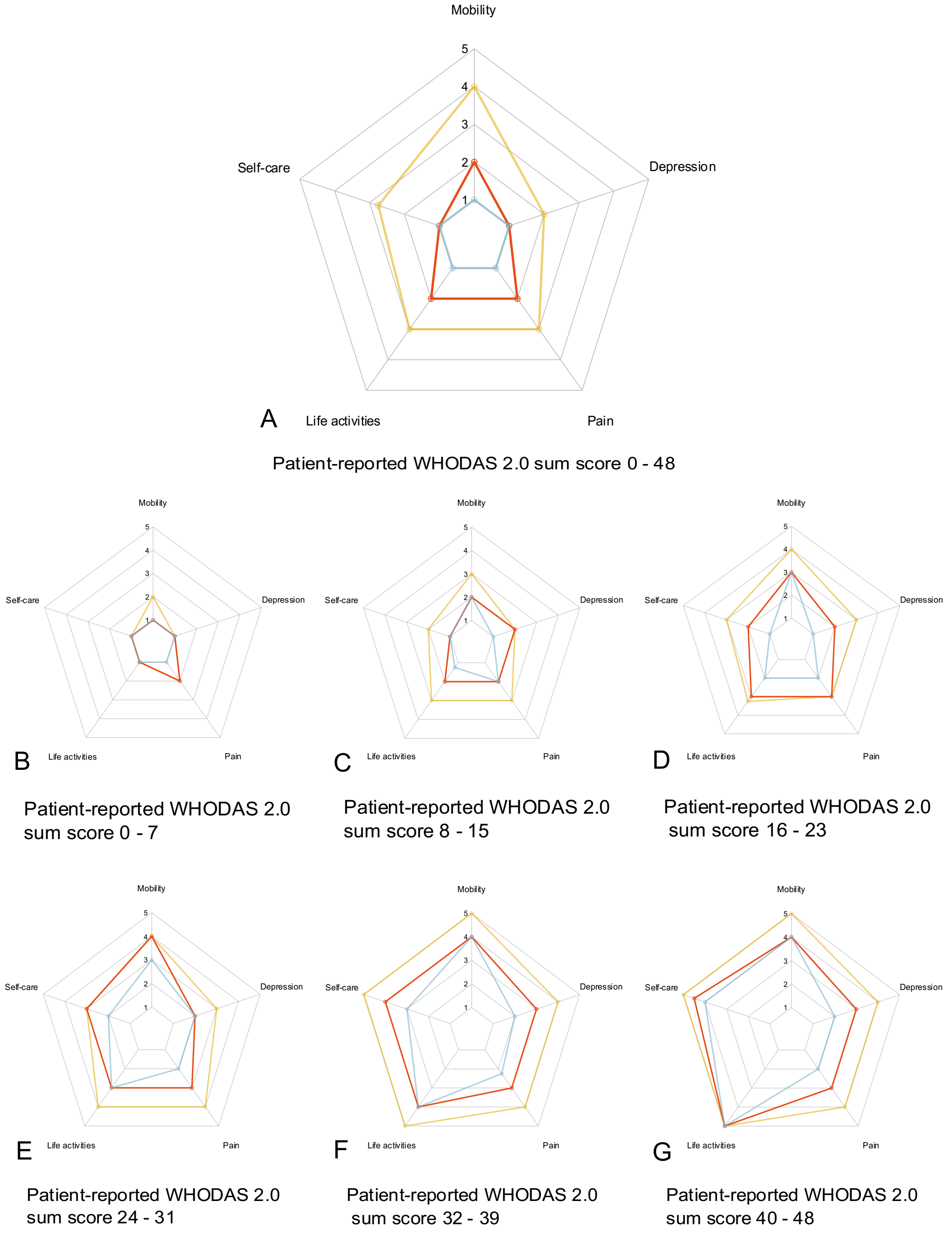


The WHODAS 2.0 sum scores range from 0 to 48 points. Each EQ-5D-5L domain has five levels, represented by the nodes of the inner circle (1) to the outer circle (5). Nodes closer to the center indicate better functioning. Blue and yellow dots and lines indicate Q1 and Q3, red dots and lines indicate the median. EQ-5D-5L, EuroQol 5-Dimensions 5-Level; WHODAS 2.0, WHO Disability Assessment Schedule 2.0.

**Figure S3.** EQ-5D-5L domain results, stratified by proxy-reported WHODAS 2.0 sum score groups.

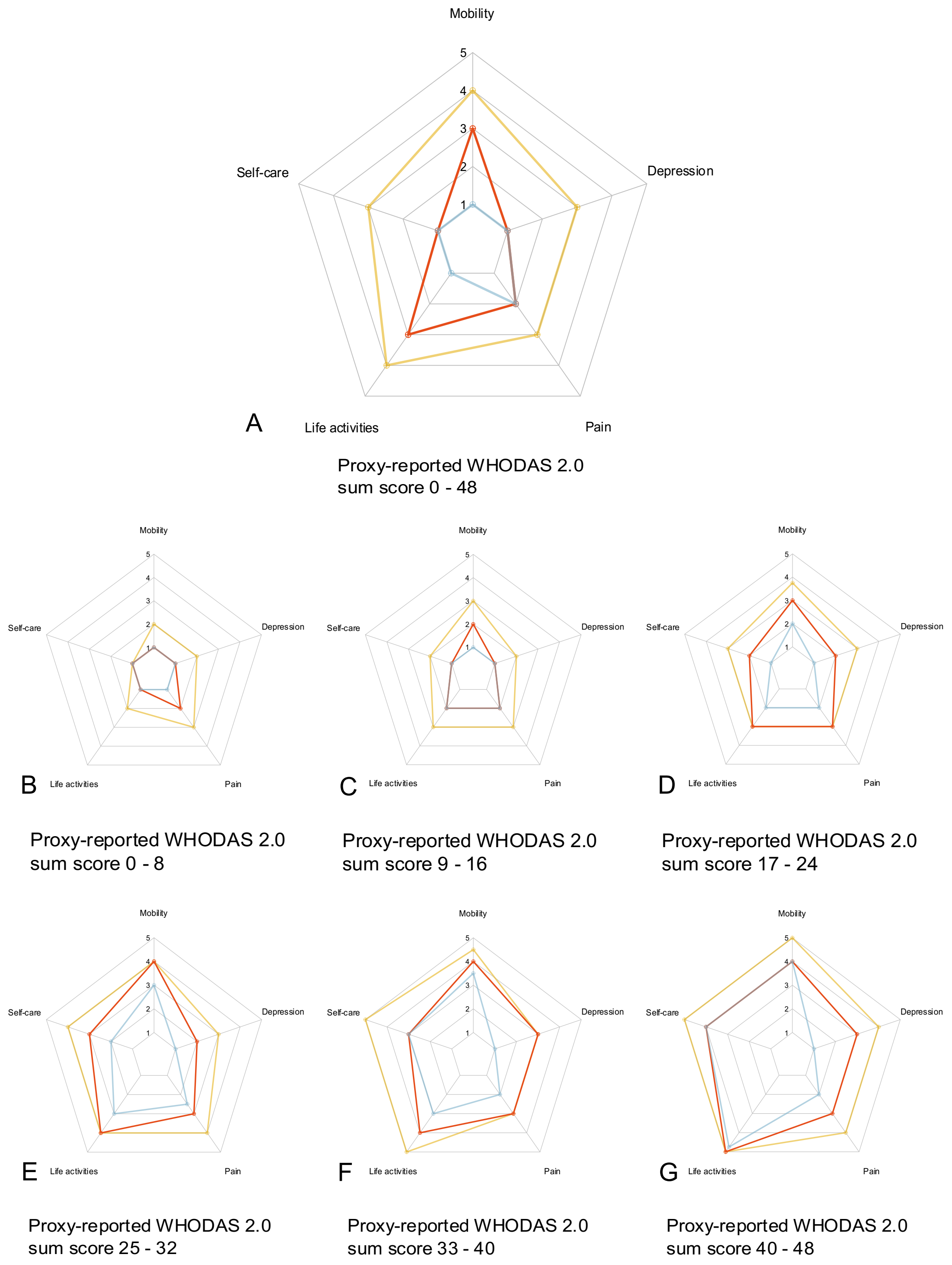


The WHODAS 2.0 sum scores range from 0 to 48 points. Each EQ-5D-5L domain has 5 levels, represented by the nodes of the inner circle (1) to the outer circle (5). Nodes closer to the center indicate better functioning. Blue and yellow dots and lines indicate Q1 and Q3, red dots and lines indicate the median. EQ-5D-5L, EuroQol 5-Dimension 5-Level; WHODAS 2.0, WHO Disability Assessment Schedule 2.0.
